# A Case of Pyometra Caused by Achromobacter xylosoxidans and γ-Streptococcus in an Elderly Frail Woman

**DOI:** 10.7759/cureus.32016

**Published:** 2022-11-29

**Authors:** Shinichi Mukai, Hiroki Isono, Keisuke Kondo, Kousuke Ihara, Momoko Isono, Haruki Ogawa

**Affiliations:** 1 General Medicine, HITO Medical Center, Ehime, JPN; 2 Obstetrics and Gynaecology, HITO Medical Center, Ehime, JPN

**Keywords:** achromobacter xylosoxidans, gynecological consultation, older woman, drainage of pus, fever, pyometra

## Abstract

Pyometra is an easily overlooked disease with nonspecific symptoms; however, a delayed diagnosis can lead to severe complications. An 80-year-old frail woman presented to our hospital with a chief complaint of persistent fever for 10 days. Her blood tests showed an elevated inflammatory response, and computed tomography showed a 10-cm cystic lesion in the pelvic floor compressing the bladder. A catheter was inserted from the vagina into the uterine cavity, resulting in pus drainage and pyometra diagnosis. A pus culture was subsequently performed, which detected *Achromobacter xylosoxidans*, a common cause of respiratory tract infections in cystic fibrosis and bloodstream infections, and*γ-streptococcus*. To the best of our knowledge, this is the first report of pyometra caused by *Achromobacter xylosoxidans*. The patient was treated with drainage and piperacillin-tazobactam administration. Pyometra is especially prevalent in older women with impaired activities of daily living and dementia. Although fever, lower abdominal pain, and increased discharge may occur, symptoms are often nonspecific, and half of such cases are asymptomatic. Furthermore, delayed diagnosis can lead to perforation of the uterus and consequent pan-peritonitis. Thus, the diagnosis of pyometra should be considered in older women presenting with unknown fever, and imaging studies and gynecological consultation should be requested promptly.

## Introduction

Pyometra is an easily overlooked disease with nonspecific symptoms; however, a delayed diagnosis can lead to severe complications. Although fever, lower abdominal pain, and increased discharge may occur, its symptoms are nonspecific, and half of such cases are asymptomatic [1]. Herein, we report an older woman presenting with a fever of unknown origin diagnosed as a pyometra case caused by Achromobacter xylosoxidans and γ-streptococcus.

## Case presentation

An 80-year-old frail woman presented to our Department of General Medicine with a chief complaint of fever persisting for 10 days. She was noted to be bedridden, suffering from severe dementia, unable to communicate, and was fully assisted in all activities of daily living. There were no significant physical findings except a high fever (38 ºC). Internal examination and transvaginal ultrasonography revealed an atrophic uterovaginal area with no erosions or masses. There were no abnormalities in the vagina, uterus, or bilateral adnexa.

**Figure 1:**
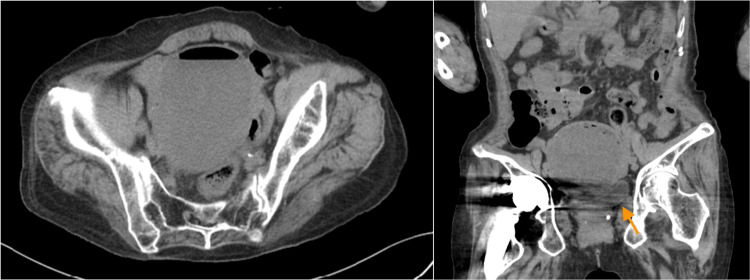
Non-contrast computed tomography of the abdomen. A 10 cm large cystic lesion with air inside is seen on the pelvic floor. It is compressing the bladder (arrow).

**Figure 2:**
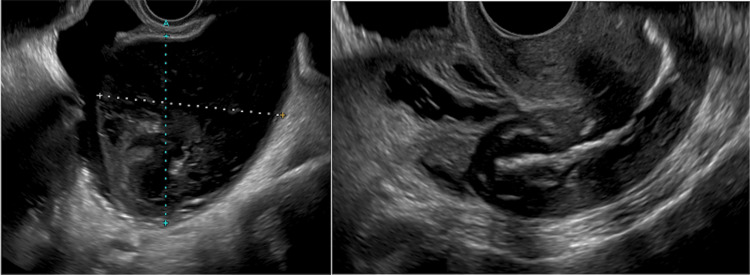
Transvaginal ultrasound Left: An enlarged uterus (77 mm x 78 mm) with internal fluid retention. Right: The uterus after drainage.

Moreover, uterine mobility was good, and no tenderness was observed. Her blood tests also showed an elevated inflammatory response, but no pyuria was noted (Table 1). Chest radiography showed no abnormalities. However, non-contrast abdominal computed tomography (CT) showed a 10-cm cystic mass with air in the pelvic floor that was compressing the bladder without any findings of ascites (Figure 1). The metal artifact of the artificial femoral head was also noted to be superimposed. In addition, a transvaginal ultrasound was performed, which revealed an enlarged uterine cavity with internal fluid retention (Figure 2). A catheter was then inserted into the uterine cavity through the vagina, which drained 430 ml of pus, leading to the diagnosis of pyometra. Pus culture was subsequently done, revealing growths of Alcaligenes xylosoxidans and γ-streptococcus. Antimicrobial susceptibility testing reported on day 7 of hospitalization showed that Alcaligenes xylosoxidans was susceptible to piperacillin, meropenem, minocycline, and sulfamethoxazole-trimethoprim (Table 2).

**Table 1:** Laboratory tests Neut: neutrophils, RBC: red blood cell, Hb: hemoglobin, PLT: platelet, AST: aspartate aminotransferase, ALT: alanine aminotransferase, γ-GTP:gamma-glutamyl transpeptidase, BUN: blood urea nitrogen, S. creatinine: serum creatinine, CRP: C-reactive protein, WBC: white blood cell.

Laboratory tests	On the day of the presentation	Day 5 of hospitalization	Reference range
Leukocyte (/µl)	25,300	7,000	3,300-8,600
Neut (%)	91	73	37-74
RBC (million/µl)	386	362	386-492
Hb (g/dl)	11.7	10.9	11.6-14.8
PLT (/µl)	393,000	420,000	158,000-348,000
AST (U/l)	27	23	7-38
ALT (U/l)	26	18	4-44
γ-GTP (U/l)	49	30	9-32
BUN (mg/dl)	14.7	9.5	8.0-20.0
S. creatinine (mg/dl)	0.46	0.54	0.46-0.79
CRP (mg/dl)	10.13	1.47	<0.14
Urinalysis results	On the day of the presentation		Reference range
WBC	1-4/HPF		<5/HPF
ketone	negative		negative

**Table 2:** Antimicrobial susceptibility testing for Alcaligenes xylosoxidans

Antimicrobial agent	Susceptibility	MIC (μg/ml)
Ampicillin	Resistant	32<
Piperacillin	Susceptible	<4
Amoxicillin / Clavulanate	Susceptible	8
Cefazolin	Resistant	64<
Cefmetazole	Resistant	64<
Cefotaxime	Resistant	64<
Cefepime	Resistant	64<
Imipenem	Susceptible	1
Merpenem	Susceptible	1
Cefotiam	Resistant	64<
Gentamicin	Resistant	16<
Amikacin	Resistant	64<
Minocycline	Susceptible	4
Ciprofloxacin	Resistant	4<
Levofloxacin	Intermediate	4
Sulfamethoxazole - Trimethoprim	Susceptible	<20

In contrast, γ-streptococcus was susceptible to all penicillins, cephems, and fluoroquinolones. Piperacillin-tazobactam was administered for seven days, with a total of three intermittent drainages of pus and intrauterine lavage at the Department of Gynecology. The patient's fever resolved promptly after treatment was initiated, and on Day 9 of hospitalization, fluid accumulation in the uterine cavity disappeared. She was discharged on Day 18 of hospitalization. Two months later, an internal gynecological examination and transvaginal ultrasound showed no accumulation of pus, and no findings suggest malignancy. Due to the patient's background of being bedridden and unable to communicate for many years, curettage of the uterine cavity and biopsy of the endometrium was not performed. Total hysterectomy was also not indicated for this patient, regardless of if a malignant tumor was found.

## Discussion

The prevalence of pyometra increases with age, affecting approximately 13% of the elderly [1]. Although it is rare in humans, pyometra is a well-known disease in veterinary medicine, with a high incidence in dogs and cats [2]. Treatment in animals is generally done by hysterectomy and ovariectomy. In human pyometra, the cause may be an organic closure of the uterine cavity; however, the incidence of gynecological malignancies complicating pyometra remains unclear. A study reported that the leading cause of pyometra is a malignancy of the genital tract system, including approximately 35% of known cases. In comparison, the remaining 65% have been attributed to other benign conditions, including benign tumors (e.g., leiomyoma, cervical polyps), infections (e.g., senile cervicitis), post-surgical cervical occlusion, radiation exposure, or forgotten intrauterine devices [3]. Contrarily, there are reports that the rate of malignancies complicating pyometra uteri is 6 out of 27 (22.2%) and 1 out of 57 (1.8%) [4,5]. Meanwhile, the risk of idiopathic pyometra is considered high, especially in elderly patients with urinary or fecal incontinence, concurrent medical conditions, or bedridden [6,7]. Therefore, although malignancy should not be overlooked, there are cases in which malignancy screening is of little significance, as in our case.

Symptoms of pyometra can include fever, lower abdominal pain, lower abdominal distention, and a feeling of tightness; however, these symptoms are often nonspecific [7]. Since pyometra more commonly affects older patients with impaired activities of daily living, it is often mistaken for a urinary tract infection and can be easily overlooked. Furthermore, half of these cases have been reported to be asymptomatic [8]. In the present case, since the source of fever could not be identified on initial examination and there was no pyuria, a CT scan was performed on the same day, which allowed for an early diagnosis. Retrospectively, after looking at the CT images, the mass may have been palpable in the abdomen, but this could not be known because the abdominal examination was not repeated. Since cystic lesions can be misdiagnosed as normal bladder even with imaging studies, they should be evaluated with pyometra in mind [1].

Pyometra diagnosis is made through evidence of pus retention in the uterine cavity. The most common causative organisms are aerobic bacteria, such as Streptococcus spp., Escherichia coli, and Enterococcus spp., and anaerobic bacteria, such as Bacteroides spp [9]. Pseudomonas aeruginosa and Mycobacterium tuberculosis have also been reported, indicating diversity in the causative organisms for pyometra [8]. To the best of our knowledge, this is the first case of pyometra by Achromobacter xylosoxidans was first isolated in Japan in 1971 from an ear discharge sample from a patient with chronic otitis media [10]. The frequent sources of Achromobacter xylosoxidans infection include respiratory tract infections in cystic fibrosis and bloodstream infections. Other reported cases include intra-abdominal, urinary tract, central nervous system, skin, soft tissue, and wound infections; mediastinitis; infective endocarditis; and intraocular and otitis media infections. Most cases are nosocomial, with device-related infections, especially intravascular catheters, being the most common [4,11,12]. In this case, no prior antibiotic administration, catheter placement, or recent hospitalization was noted; thus, we did not know the predisposing cause for our patient. In addition, γ-streptococcus, which was simultaneously detected, is a joint causative agent of pyometra. The extent to which Achromobacter xylosoxidans and γ-streptococcus each contributed to the development of pyometra is unknown. Since the patient in our case was a long-term in-patient with pyometra, piperacillin-tazobactam was selected to cover Pseudomonas aeruginosa. Achromobacter xylosoxidans are naturally resistant to aminoglycosides, third-generation cephalosporins or lower (except ceftazidime), and aminopenicillin; treatment options include treatment options piperacillin, piperacillin-tazobactam, meropenem, imipenem, ceftazidime, and ST combinations [11].

The initial treatment of pyometra consists of dilatation, drainage of the uterine cavity, and administration of intravenous antibiotics. However, due to the high risk of relapse in some instances, surgery (hysterectomy) may be considered. In a previous study of 41 cases from Japan 30 years ago, all patients underwent dilatation and drainage of the uterine cavity; antibiotics were administered in only six cases. Except for one case in which the patient developed pelvic peritonitis and died, the remaining 40 patients have been cured [7]. In another study of 57 cases, 14% were treated with drainage but without antibiotics. The duration of antibiotic therapy is unknown [5]. No other reviews summarizing non-perforated pyometra could be found, and the appropriate duration of antibiotic administration remains unclear. In this case, the patient's fever resolved spontaneously after admission, and the abscess disappeared with drainage. Antibiotics were then discontinued in 7 days. Given all these findings, pyometra should be considered in older women with a fever of unknown origin. Imaging studies and gynecological consultation should be requested promptly, as a delayed diagnosis can lead to pan-peritonitis and septic shock due to perforation of the uterus [13,14].

## Conclusions

Herein, we reported a case of pyometra in a frail older woman with persistent fever. To the best of our knowledge, this is the first case of pyometra caused by mixed infection with Achromobacter xylosoxidans and γ-streptococcus. Pyometra is a common disease in bedridden older patients and those with dementia, and it is likely to be encountered with increasing frequency in hyper-aged society. Although it is a disease with nonspecific symptoms, delayed diagnosis and treatment may lead to severe outcomes. As such, pyometra should be a differential diagnosis in frail older women with unknown fever.
